# Emodin ameliorates renal injury and fibrosis *via* regulating the miR-490-3p/HMGA2 axis

**DOI:** 10.3389/fphar.2023.1042093

**Published:** 2023-03-03

**Authors:** Liulin Wang, Xuerui Wang, Gang Li, Shanshan Zhou, Rui Wang, Qi Long, Min Wang, Liang Li, Hai Huang, Yuanming Ba

**Affiliations:** ^1^ Hubei Provincial Hospital of Tranditional Chinese Medicine, Wuhan, China; ^2^ Affiliated Hospital of Hubei University of Traditional Chinese Medicine, Wuhan, China; ^3^ Hubei Provincial Academy of Traditional Chinese Medicine, Wuhan, China; ^4^ Beijing Hospital of Traditional Chinese Medicine, Capital Medical University, Beijing, China; ^5^ Beijing Key Laboratory of Basic Research With Traditional Chinese Medicine on Infectious Diseases, Beijing, China; ^6^ Beijing Institute of Chinese Medicine, Beijing, China

**Keywords:** emodin, renal fibrosis, microRNA, inflammatory factor, HMGA2

## Abstract

Renal fibrosis is a major pathological feature of chronic kidney disease (CKD). While emodin is reported to elicit anti-fibrotic effects on renal injury, little is known about its effects on microRNA (miRNA)-modulated mechanisms in renal fibrosis. In this study, we established a unilateral ureteral obstruction (UUO) model and a transforming growth factor (TGF)-β1-induced normal rat renal tubular epithelial cell line (NRK-52E) model to investigate the protective effects of emodin on renal fibrosis and its miRNA/target gene mechanisms. Dual-luciferase assay was performed to confirm the direct binding of miRNA and target genes in HEK293 cells. Results showed that oral administration of emodin significantly ameliorated the loss of body weight and the increase in physicochemical parameters, including serum uric acid, creatinine, and urea nitrogen in UUO mice. Inflammatory cytokines, including tumor necrosis factor-α, monocyte chemoattractant protein-1, and interleukin (IL)-1β, but not IL-6, were down-regulated by emodin administration. Emodin decreased the expression levels of TGF-β1 and fibrotic-related proteins, including alpha-smooth muscle actin, Collagen IV, and Fibronectin, and increased the expression of E-cadherin. Furthermore, miR-490-3p was decreased in UUO mice and negatively correlated with increased expression of high migration protein A2 (HMGA2). We further confirmed HMGA2 was the target of miR-490-3p. Transfection of miR-490-3p mimics decreased, while transfection of miR-490-3p inhibitors increased fibrotic-related proteins and HMGA2 expression levels in TGF-β1-induced NRK-52E cells. Furthermore, transfection of miR-490-3p mimics enhanced the anti-fibrotic effects of emodin, while transfection of miR-490-3p inhibitors abolished the protective effects of emodin. Thus, as a novel target of emodin that prevents renal fibrosis in the HMGA2-dependent signaling pathway, miR-490-3p has potential implications in CKD pathology.

## 1 Introduction

Chronic kidney disease (CKD) involves sustained damage of the renal parenchyma and chronic deterioration of renal function (Akchurin, 2019). The global burden of CKD is a major public health problem, especially in high-income countries (Gaitonde et al., 2017), with approximately 10% of adults worldwide affected by some form of CKD, resulting in 12 million deaths each year (Xie et al., 2018; Collaboration, 2020). Although there are many known causes of CKD, including diabetes mellitus, glomerulonephritis, and cystic kidney disease, the etiology of CKD remains incompletely understood (Kalantar-Zadeh et al., 2021). At present, CKD treatment primarily involves slowing disease progression and preventing complications by managing modifiable risk factors, such as diabetes and blood pressure, and initiating nephroprotective drug regimens in the early stages of disease (Curtis and Komenda, 2020). Thus, the discovery of novel therapeutic agents that can prevent or delay the progression of disease will contribute to CKD treatment.

Increasing evidence suggests that renal fibrosis is a common final stage of CKD (Klinkhammer et al., 2017). Similar to wound healing, renal fibrosis is a dynamic process with early beneficial effects on injury, but can lead to persistent pathological fibrosis, glomerulosclerosis, tubular atrophy and dilatation, and tubulointerstitial fibrosis (Boor et al., 2010; Djudjaj and Boor, 2019). Over the past 2 decades, microRNAs (miRNAs) have been implicated in fibrosis of various organs, and their role in renal fibrosis has attracted widespread attention, with new therapeutic strategies targeting miRNAs showing considerable promise (Van der Hauwaert et al., 2015; Fan et al., 2020). Transforming growth factor (TGF)-β is a key driver of renal fibrosis and is involved in the dynamic pathophysiological processes leading to CKD and end-stage renal disease (ESRD) (Ma and Meng, 2019). Recent evidence indicates that multiple miRNAs are involved in renal fibrosis through the TGF-β pathway (Zhong et al., 2013; Sun et al., 2018; Sun et al., 2021). For example, MiR-490-3p is known to play a key regulatory role in silica-induced pulmonary fibrosis (Cheng et al., 2021). Studies have also found that miR-490 is decreased in unilateral ureteral obstruction (UUO) model mice (Chung et al., 2010; Qin et al., 2011), and elevated in the urine of active segmental glomerulonephritis patients (Zhang et al., 2014). However, while miR-490 appears to play a crucial role in the pathogenesis of CKD, little is known regarding its functional role.

Emodin is a naturally occurring anthraquinone derivative and active ingredient of rhubarb (Liu et al., 2016), with diverse biological and pharmacological properties, including anticancer, anti-inflammatory, antioxidant, antibacterial, antiviral, anti-diabetic, immunosuppressive, and renoprotective activities (Dong et al., 2016). Emodin is reported to suppress renal fibrosis *via* multiple mechanisms (Ma et al., 2018; Yang et al., 2020; Liu et al., 2021), and is involved in the regulation of miRNAs in several diseases (Xiang et al., 2017; Cao et al., 2019; Xie et al., 2019). We previously reported that a stewed rhubarb decoction mitigated chronic renal failure progression in mice (Wang et al., 2022). However, further experimental research is needed to explore the potential fibrotic effects of emodin on CKD. Here, we hypothesized that miR-490-3p may be involved in the pathological process of renal fibrosis in CKD and that emodin may exert anti-fibrotic effects on CKD *via* regulation of miR-490-3p.

## 2 Materials and methods

### 2.1 Animals

Male C57BL/6 mice (age 8–10 weeks, weight 20–24 g) were purchased from Beijing Huafukang Biotechnology Co., Ltd. (Beijing, China). The mice were maintained in an environmentally controlled rearing room (temperature: 22°C ± 2°C, humidity: 50% ± 5%, 12-h light/dark cycle) for 1 week of adaptive feeding, with distilled water and sterilized food freely available. The experimental protocols used in this study were approved by the Ethics Committee for Animal Experimentation of the Hubei Provincial Hospital of Traditional Chinese Medicine (NO. 2019008) and were conducted according to the Guidelines for Animal Experimentation of the Hubei Provincial Hospital of Traditional Chinese Medicine.

### 2.2 UUO model

The UUO mouse model was established as described previously (Zhou et al., 2020). Briefly, mice were anesthetized with pentobarbital sodium (40 mg/kg body weight) by an intraperitoneal injection. The left ureter was isolated through a median incision and ligated at two points with 5–0 silk. Mice in the sham-operated control group received the same operation, except the ureter was not ligated or cut.

### 2.3 Treatment protocols

Emodin (Shanghai Yuanye Bio-Technology Co., Ltd. China) was dissolved in pyrogen-free saline and sodium carboxymethyl cellulose (Beijing ITA Biotechnology Co., Ltd., China). Daily oral emodin treatment was started 2 h after UUO surgery for 14 continuous days, with 10 mg/kg, 20 mg/kg, and 40 mg/kg considered as low (EM-L), medium (EM-M), and high (EM-H) dosages. Losartan (LST, Merck Sharp and Dohme., Ltd., United States) was dissolved in pyrogen-free saline as a positive control. Daily oral LST treatment (10 mg/kg) was started 2 h after UUO surgery for 14 continuous days. All sham and UUO mice were treated with the same volume of saline and sodium carboxymethyl cellulose as the vehicle for treatment.

### 2.4 Cell culture and treatment

The normal rat renal tubular epithelial cell line NRK-52E was purchased from American Type Culture Collection (ATCC, Manassas, United States), and routinely cultured in Dulbecco’s modified Eagle’s medium (DMEM, Wisent, Nanjing, China) containing 10% fetal bovine serum (Gibco, Gaithersburg, MD, United States), 100 U/ml penicillin (Gibco, Carlsbad, CA, United States), and 100 μg/mL streptomycin (Gibco, Carlsbad, CA, United States) at 37°C with 5% CO_2_. In the first experiment, NRK-52E cells were pretreated with serum-free medium for 24 h, then exposed to 40 μM or 80 μM emodin (Shanghai Yuanye Bio-Technology Co., Ltd., China) for 24 h. In the second experiment, NRK-52E cells were pretreated with serum-free medium for 24 h, then exposed to 10 ng/mL TGF-β1 (R&D Systems, MN, United States) with or without 40 μM or 80 μM emodin for 24 h. In the third experiment, HEK293 cells were transfected with miR-490-3p mimics, miR-490-3p inhibitors, and negative controls (NCs), then treated with or without 10 ng/mL TGF-β1 for 24 h. In the fouth experiment, NRK-52E cells transfected with miR-490-3p mimics, miR-490-3p inhibitors, and NCs were treated with or without 80 μM emodin for 24 h, then exposed to 10 ng/mL TGF-β1 for 24 h. All control groups were treated with the same volume of vehicle.

### 2.5 Transfection of miRNA mimics and inhibitors

The miR-490-3p mimics (5'-CAA​CCU​GGA​GGA​CUC​CAU​GCU​G-3'), inhibitors (5'-AUU​CGU​CCU​CCU​GAC​CAU​GGU​C-3'), and NCs were obtained from Biomics Biotech (Nantong, China). The NRK-52E cells were transfected with 50 nM miR-490-3p mimics or inhibitors, and corresponding NCs at the same concentration, using Liposome 2000 reagent (Invitrogen, United States) according to the manufacturer’s instructions. Cells were collected 24 h after transfection for further experimental analysis.

### 2.6 Luciferase reporter assay

HEK293 cells were co-transfected with NCs or miR-490-3p mimics (50 nM), CMV-Renilla, HMGA2 promoter-luciferase using Lipofectamine 2000 reagent (Invitrogen, United States) following the manufacturer’s protocols. The plasmids were constructed and provided by Hunan Fenghui Biotechnology Co., Ltd. (Cat. BR082). Luciferase activities were measured 48 h after transfection using dual-luciferase assay kits according to the manufacturer’s protocols (Promega, United States).

### 2.7 Cell counting Kit-8 (CCK-8) assay

The cytotoxicity of emodin was determined by CCK-8 assay. Cells were seeded into 96-well plates. According to the instructions of the CCK-8 kit (Beyotime, Shanghai, China), each well was supplied with 10 μL of CCK-8 after treatment, then incubated at 37°C for 4 h. Absorbance was recorded at 450 nm, and experiments were performed in parallel and in triplicate.

### 2.8 Physiological and biochemical parameters

Body weights were recorded on day 14 after UUO surgery. Plasma creatinine, urea, and uric acid levels were detected using a creatinine colorimetric kit, urea colorimetric kit, and uric acid colorimetric kit (Bohu Biotechnology Co., Ltd. Shanghai, China), respectively, according to the manufacturer’s instructions.

### 2.9 Histological examination

Renal tissues were fixed in 4% formaldehyde, dehydrated, embedded, and sectioned at 5 μm thickness. Renal sections were stained with hematoxylin and eosin (H&E) or Masson’s trichrome staining (Solarbio Life Sciences, Beijing, China) according to the manufacturer’s instructions. Sections were observed and scored in a blinded manner. Tubular injury scores based on morphological damage (epithelial necrosis, tubular necrotic debris, and tubular dilatation) were quantified in three or four sections and 10–12 regions per kidney, with a score range of 0–4 (0: 0%; 1: <25%; 2: 26%–50%; 3: 51%–75%; 4: ≥76% of renal tubular injury) (Liu N. et al., 2015; Ren et al., 2021). All positive signals from the histological images (at least three areas per sample) were quantified using ImageJ v1.8.0 software (National Institutes of Health, Bethesda, MD, United States).

### 2.10 Enzyme-linked immunosorbent assay (ELISA)

Mouse blood was collected from the abdominal aorta 14 days after UUO surgery and centrifuged at 3,000 r/min for 15 min. Serum was collected and stored in a −80°C refrigerator for testing. The levels of interleukin (IL)-1β, tumor necrosis factor-α (TNF-α), IL-6, and monocyte chemoattractant protein-1 (MCP-1) were detected using ELISA kits (ABclonal Biotechnology Co., Ltd. China) according to the manufacturer’s protocols.

### 2.11 Western blot analysis

Total proteins were isolated from kidney tissues and cells using RIPA lysis and extraction buffer (Biyuntian, Shanghai, China). Protein content was determined using BCA protein assay reagent (Thermo, Rockford, IL, United States). The proteins were separated by sodium dodecyl sulfate polyacrylamide gel electrophoresis (SDS-PAGE) and transferred to polyvinylidene fluoride (PVDF) membranes. The membranes were blocked in 5% skim milk in phosphate-buffered saline-Tween at room temperature for 1 h and probed with specific antibodies overnight at 4°C. Primary antibodies included alpha-smooth muscle actin (α-SMA) (1:1,000, Abcam, United Kingdom), Collagen IV (1:1,000, Abcam, United Kingdom), Fibronectin (1:1,000, Abcam, United Kingdom), E-cadherin (1:1,000, Abcam, UK), TGF-β1 (1:1,000, Abcam, United Kingdom), HMGA2 (1:1,000, Invitrogen, United States), and β-actin (1:1,000, Boster, China). Horseradish peroxidase-conjugated secondary antibodies (Sigma, United States) were used for detection of specific proteins for an additional 60 min β-actin was used as the loading control. After a final wash with tris-buffer physiological saline (TBST), immunoreactive bands were detected using an Odyssey Imaging Analyzer (LAS-4000; Toyobo Engineering, Osaka, Japan). Signal intensity was measured using ImageJ and normalized to β-actin signal intensity.

### 2.12 RNA extraction and real-time polymerase chain reaction (Real-time PCR)

Total RNA from mouse renal tissues, HEK293 cells, and NRK-52E cells was extracted using Trizol Reagent (Invitrogen) according to the manufacturer’s instructions. For miRNA, total RNA was reverse transcribed into cDNA using a microRNA First-Strand cDNA Synthesis Kit (Sangong Biotech, Shanghai, China). For mRNA, cDNA was obtained using the GoScript Reverse Transcription System Kit (Promega, Madison, WI, United States). GAPDH and small nuclear U6 were used as endogenous controls for mRNA and miRNA levels respectively. Gene expression levels were analyzed by Real-time PCR performed using 2 × SYBR master mix (Takara, Otsu, Shiga, Japan) and a BioRad iCycler iQ5 (BioRad, Hercules, CA, United States). GAPDH and U6 were used as the endogenous controls for measurement of mRNA expression level and miRNA expression analysis, relative mRNA expressions were compared with normalized Sham group. All samples were run in duplicate. Primer sequences are listed in Table 1.

**TABLE 1:** The sequences of the primers.

Name	Sequences
miR-490-3p	Forward: 5'-AAC​ACG​TGC​AAC​CTG​GAG​GAC-3'
	Reverse: 5'-ATC​CAG​TGC​AGG​GTC​CGA​GG-3’
U6	Forward: 5′-GCT​TCG​GCA​GCA​CAT​ATA​CTA​AAA​T-3′
	Reverse: 5′-GCT​TCG​GCA​GCA​CAT​ATA​CTA​AAA​T-3′
mus-HMGA2	Forward: 5'-CCT​AAG​AGA​CCC​AGA​GGA​AGA-3'
	Reverse: 5'- CGA​CTT​GTT​GTG​GCC​ATT​TC-3'
homo-HMGA2	Forward: 5'- GTC​CCT​CTA​AAG​CAG​CTC​AAA-3'
	Reverse: 5'- TGA​GCA​GGC​TTC​TTC​TGA​AC-3'
rattus-HMGA2	Forward: 5'- CTG​GAC​GTC​CGG​TGT​TGG​T-3’
	Reverse: 5’-AAC​ACC​TTT​CGG​GAG​ACG​GG-3’
mus-MCP-1	Forward: 5'-TTA​AAA​ACC​TGG​ATC​GGA​ACC​AA-3'
	Reverse: 5'-GCA​TTA​GCT​TCA​GAT​TTA​CGG​GT-3'
mus-TNFα	Forward: 5'-ACC​CTC​ACA​CTC​AGA​TCA​TCT​TC-3'
	Reverse: 5'-TGG​TGG​TTT​GCT​ACG​ACG​T-3'
mus-IL-6	Forward: 5'-ACA​AAG​CCA​GAG​TCC​TTC​AGA​GA-3'
	Reverse: 5'-CTG​TTA​GGA​GAG​CAT​TGG​AAA​TTG-3'
mus-IL-1β	Forward: 5'-TGG​CAA​CTG​TTC​CTG-3'
	Reverse: 5'-GGA​AGC​AGC​CCT​TCA​TCT​TT -3'
mus-GAPDH	Forward: 5'- TGT​GTC​CGT​CGT​GGA​TCT​GA-3'
	Reverse: 5'- CCT​GCT​TCA​CCA​CCT​TCT​TGA​T-3'
homo-GAPDH	Forward: 5'- CTG​CCA​ACG​TGT​CAG​TGG​TG-3'
	Reverse: 5'- GTC​GCT​GTT​GAA​GTC​AGA​GGA​G-3'
rattus-GAPDH	Forward:5’-ACAGTCCATGCCATCACTGCC-3’
	Reverse:5’-GCCTGCTTCACCACCTTCTTG-3’

### 2.13 Statistical analysis

All experimental data were analyzed using SPSS v20.0 and expressed as mean ± standard deviation (x ± s). One-way analysis of variance (ANOVA) was used for comparisons between multiple groups, and *p < 0.05* was considered statistically significant. All data in the manuscript were analyzed by an investigator blind to the experimental groups.

## 3 Results

### 3.1 Emodin ameliorates the physiological, biochemical and pathological damage in UUO mice

**FIGURE 1:**
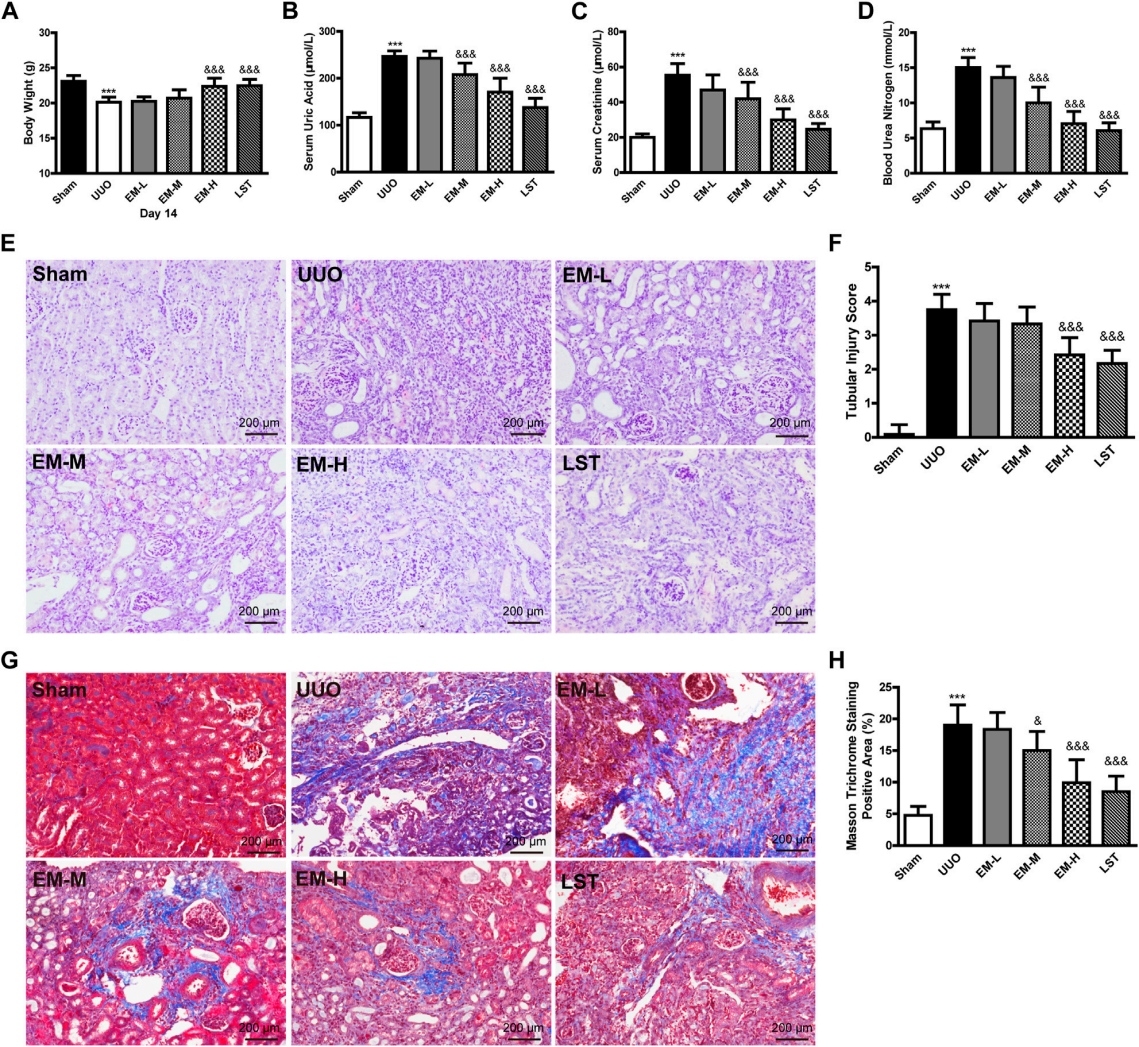
Emodin ameliorates the physiological, biochemical and pathological damage in UUO mice. Mice were randomly divided into Sham, UUO, EM-L, EM-M, EM-H, and LST groups. Mice were administrated with dosages of 10 mg/kg, 20 mg/kg, 40 mg/kg emodin, or 10 mg/kg Losartan started 2 h after UUO surgery, daily, orally, for 14 continuous days. **(A)** Body weight, **(B)** levels of uric acid, **(C)** levels of creatinine, and **(D)** levels of urea nitrogen were detected at day 14. Representative pictures of renal **(E)** H&E and **(G)** masson staining. Scale bar = 200 μm. **(F)** Tubule injury index score and **(H)** masson trichromatic positive area were calculated at day 14. Data were shown as mean ± SD (n = 10 per group). *compared with Sham, ^*&*^compared with UUO, *p < 0.05, **p < 0.01,***p < 0.001 of each symbol, respectively. ^*&*^ *p <* 0.05, ^*&&*^ *p <* 0.01, ^*&&&*^ *p <* 0.001 of each symbol, respectively. UUO, unilateral ureteral obstruction; EM-L, emodin low dosage; EM-M, the emodin medium dosage, EM-H, emodin high dosage; LST, Losartan.

As shown in Figure 1, after 14 days of treatment, significant differences in body weight and creatinine, urea nitrogen, and uric acid levels were observed between the UUO and Sham groups. Data showed that body weight in the UUO group was significantly lower compared to the Sham group, while body weight in the EM-H group was significantly higher compared to the UUO group (Figure 1A). In addition, EM-L, EM-M, and EM-H treatment reduced creatinine, urea nitrogen, and uric acid levels to varying degrees in the UUO mice (Figures 1B–D), with emodin at 40 mg/kg showing the strongest modulatory effect.

We used H&E and Masson’s trichrome staining to evaluate renal damage and fibrosis. Glomeruli and tubules in the Sham group were structurally normal, with closely aligned tubules. However, kidneys in the UUO mice exhibited severe structural disorder, inflammatory cell infiltration, tubular atrophy, and necrosis (Figures 1E,F). Based on Masson’s trichrome staining, banded interstitial fibrosis and collagen fiber hyperplasia were observed in the UUO mice (Figures 1G,H). In contrast, treatment with emodin and LST alleviated these pathological changes in the UUO mice (Figures 1E–H).

### 3.2 Emodin reduces inflammatory cytokines release in UUO mice

**FIGURE 2:**
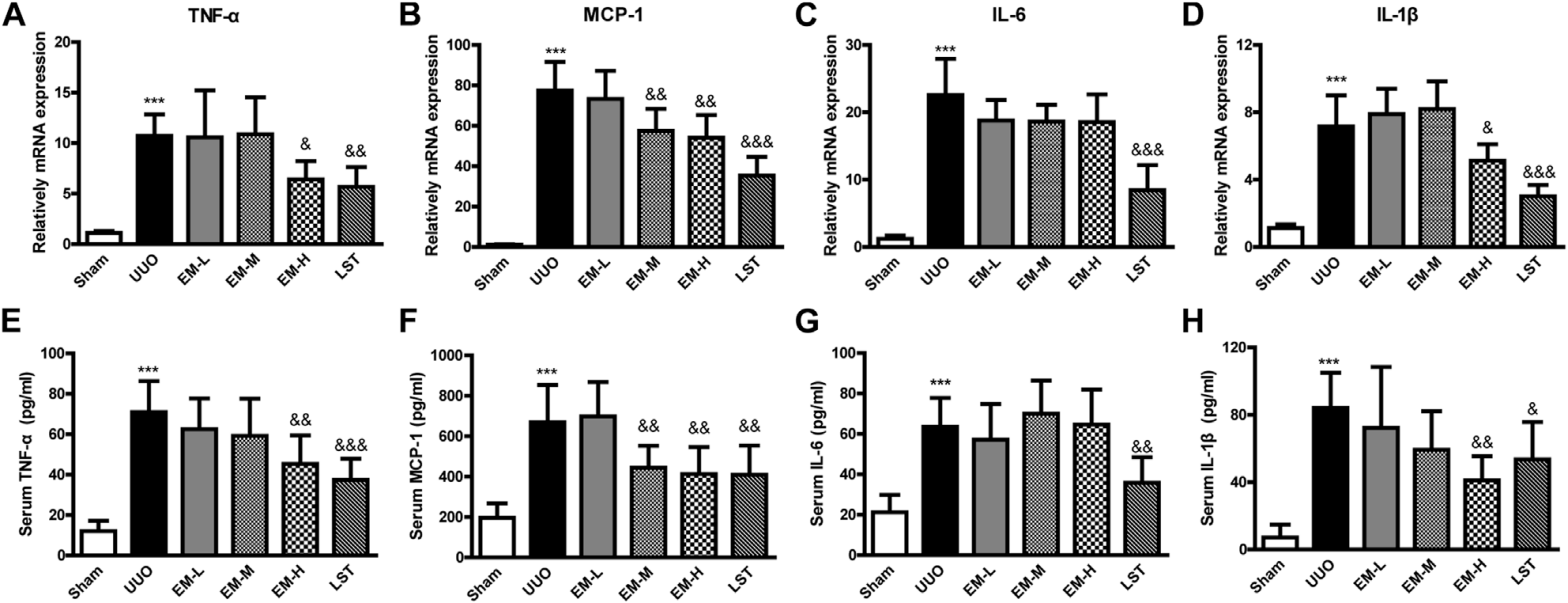
Emodin reduces inflammatory cytokines release in UUO mice. Mice were randomly divided into Sham, UUO, EM-L, EM-M, EM-H and LST groups. Mice were administrated with dosages of 10 mg/kg, 20 mg/kg, 40 mg/kg emodin, or 10 mg/kg Losartan started 2 h after UUO surgery, daily, orally, for 14 continuous days. Gene levels of **(A)** TNF-α, **(B)** MCP-1, **(C)** IL-6, and **(D)** IL-1β in renal tissues were determined by Real-time PCR. Serum levels of **(E)** TNF-α, **(F)** MCP-1, **(G)** IL-6, and **(H)** IL-1β were determined by ELISA. Data were shown as mean ± SD (n = 10 per group). *compared with Sham, ^*&*^compared with UUO, *p < 0.05, **p < 0.01,***p < 0.001 of each symbol, respectively. ^*&*^ *p <* 0.05, ^*&&*^ *p <* 0.01, ^*&&&*^ *p <* 0.001 of each symbol, respectively. UUO, unilateral ureteral obstruction; EM-L, emodin low dosage; EM-M, emodin medium dosage, EM-H, emodin high dosage; LST, Losartan; TNF-α, tumor necrosis factor-α; MCP-1, monocyte chemoattractant protein-1; IL-6, interleukin-6; IL-1β, interleukin-1β.

To further investigate the effects of emodin, we used Real-time PCR and ELISA to detect the inflammatory factors TNF-α, MCP-1, IL-6, and IL-1β in UUO mice. Compared with the Sham group, inflammatory factors in the UUO group increased significantly, after emodin intervention, TNF-α, MCP-1 and IL-1β decreased significantly in the UUO mice, while IL-6 showed a non-significant decrease (Figures 2A–F). These data indicated that emodin can reduce inflammation in UUO mice.

### 3.3 Emodin regulates fibrotic related protein expressions in UUO mice

**FIGURE 3:**
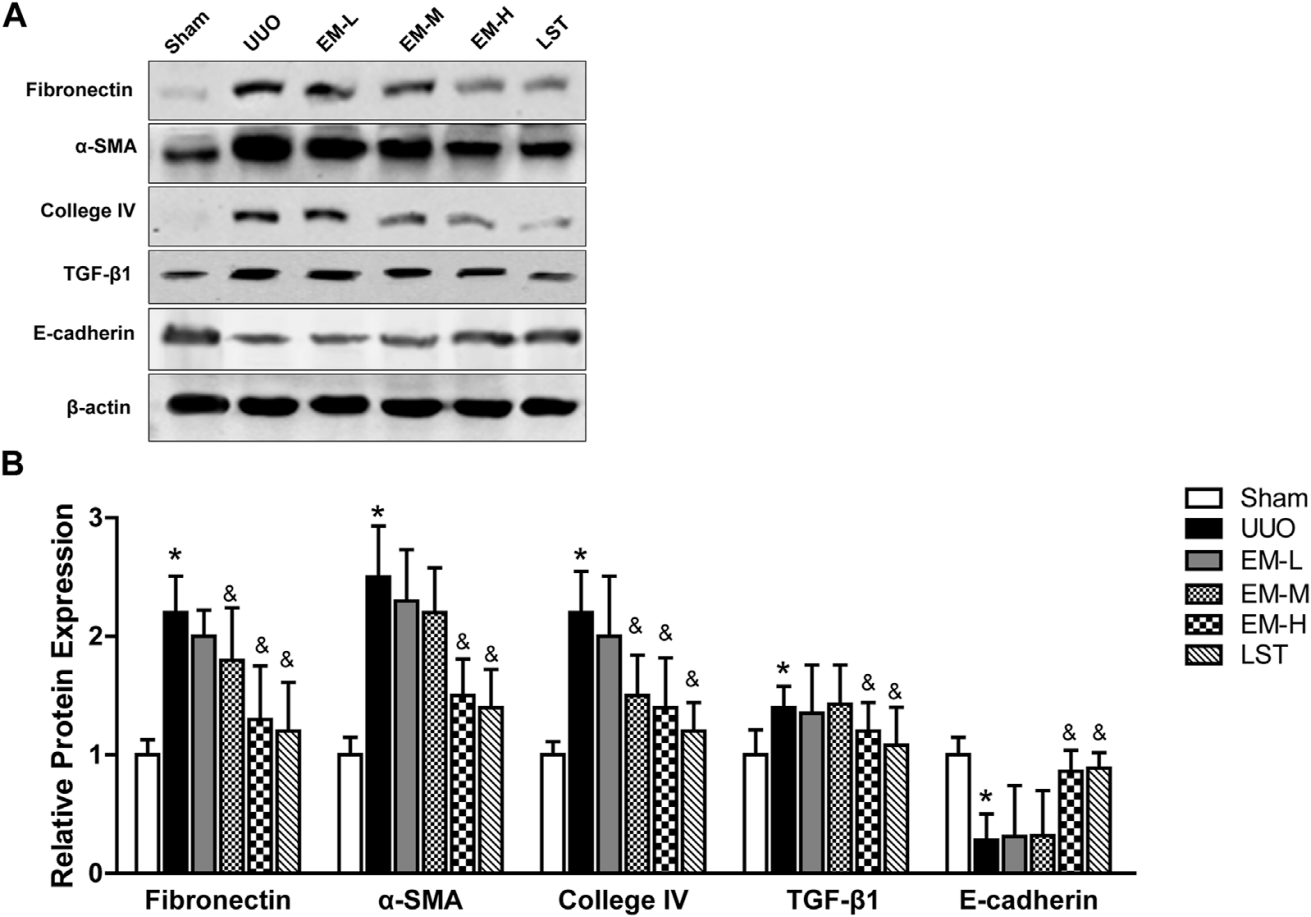
Emodin regulates fibrotic related protein expressions in UUO mice. Mice were randomly divided into Sham, UUO, EM-L, EM-M, EM-H and LST groups. Mice were administrated with dosages of 10 mg/kg, 20 mg/kg, 40 mg/kg emodin, or 10 mg/kg Losartan started 2 h after UUO surgery, daily, orally, for 14 continuous days. Representative blots of Fibronectin, α-SMA, Collagen IV,TGF-β1, E-cadherin and β-actin in the renal tissues, and **(B)** statistical analyses of relative protein expressions. Data were shown as mean ± SD (n = 10 per group). ^*^ *p < 0.05* vs. Sham, ^*&*^ *p < 0.05* vs. UUO. UUO, unilateral ureteral obstruction; EM-L, emodin low dosage; EM-M, emodin medium dosage, EM-H, emodin high dosage; LST, Losartan. TGF-β1, transforming growth factor-β1; α-SMA, alpha-smooth muscle protein.

We used western blot analysis to fibrotic related protein expressions. The protein expression levels of Fbronectin, α-SMA, Collagen IV, and TGF-β1 increased, while that of E-cadherin decreased significantly in the UUO mice. In contrast, emodin decreased Fbronectin, α-SMA, Collagen IV, and TGF-β1 expression and increased E-cadherin expression (Figures 3A,B), suggesting that emodin can reduce fibrosis in UUO mice.

### 3.4 Decreased miR-490-3p expression and increased HMGA2 expression are negatively correlated in UUO mice

**FIGURE 4:**
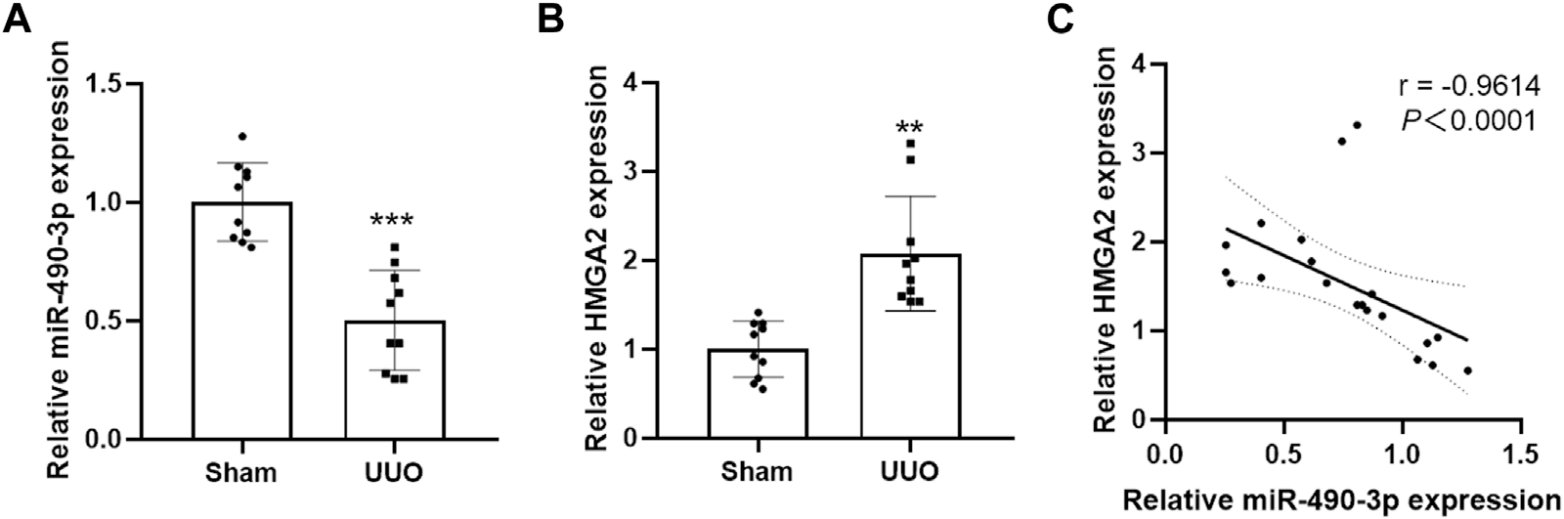
Decreased miR-490-3p expression and increased HMGA2 expression are negatively correlated in UUO mice. Gene Levels of **(A)** miR-490-3p, and **(B)** HMGA2 were detected by Real-Time PCR. **(C)** A negative correlation were found between miR-490-3p and HMGA2 mRNA expressins in the renal tissues of Sham and UUO mice (Spearman correlation analysis, r = −0.9614, *p < 0.0001*). Data were shown as mean ± SD (n = 10 per group). ^**^ *p < 0.01* vs. Sham, ^***^ *p < 0.001* vs. Sham. UUO, unilateral ureteral obstruction; HMGA2, high mobility protein A2.

We compared the endogenous expression levels of miR-490-3p and HMGA2 in the kidney tissues of male C57BL/6 mice in the Sham and UUO groups by Real-time PCR. As shown in Figure 4A, miR-490-3p expression was significantly lower in the kidneys of the UUO group than the matched Sham group. However, HMGA2 mRNA expression was increased (Figure 4B), and miR-490-3p expression was negatively correlated with HMGA2 expression (Figure 4C), further suggesting that miR-490-3p and HMG2A may be associated with pathological injury in UUO mice.

### 3.5 Emodin reversed the fibrosis in TGF-β1- induced NRK-52E cells

**FIGURE 5:**
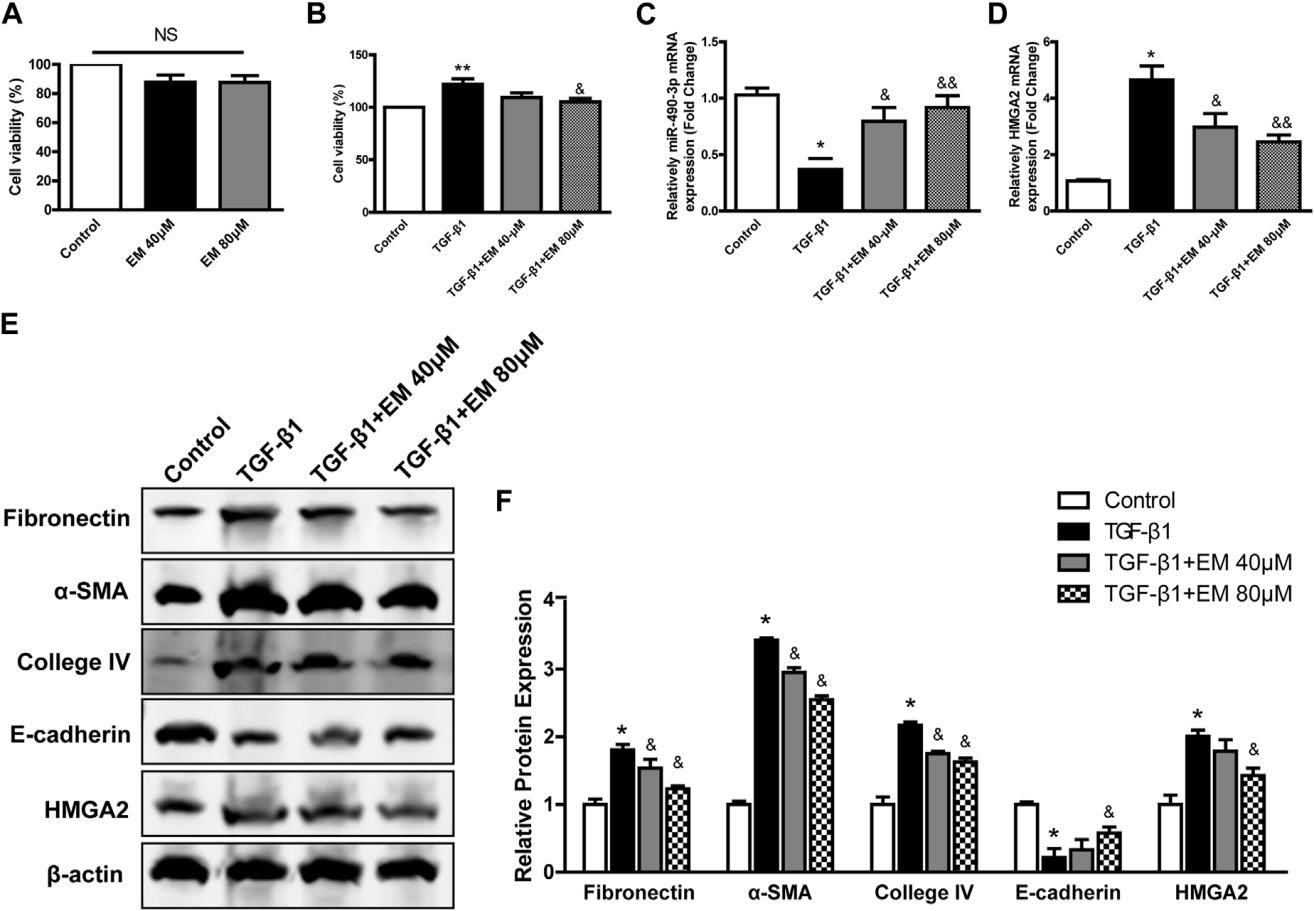
Emodin reversed the fibrosis in TGF-β1- induced NRK-52E cells. CCK-8 assay were performed in emodin-treated NRK-52E cells **(A)** without or **(B)** with TGF-β1 stimulation. The gene levels of **(C)** miR-490-3p and **(D)** HMGA2 were evaluated by Real-Time PCR. **(E)** Representative blots of Fibronectin, α-SMA, Collagen IV, E-cadherin, and HMGA2 in NRK-52E cells, and **(F)** statistical analyses of relative protein expressions. Data were shown as mean ± SD (n = 6). *compared with control, ^&^compared with TGF-β1. *p < 0.05, **p < 0.01 of each symbol, respectively. ^*&*^ *p <* 0.05, ^*&&*^ *p <* 0.01 of each symbol, respectively. TGF-β1, transforming growth factor-β1; HMGA2, high mobility protein A2; α-SMA, alpha-smooth muscle protein.

To investigate the effects of emodin on NRK-52E cell fibrosis, we first investigated the effects of emodin on NRK-52E cell viability using CCK-8. We found that different concentrations of emodin (40 and 80 μM) had no effect on cell viability (Figure 5A). As shown in Figure 5B, cell viability increased significantly in the TGF-β1 group, and decreased in the TGF-β1-induced NRK-52E cells in the emodin group (80 μM).

We next investigated the effects of emodin on renal fibrosis *in vitro* using western blot analysis to detect fibrosis-related protein levels. Compared to the TGF-β1 group, Fibronectin, α-SMA, Collagen IV decreased, while epithelial marker E-cadherin increased after emodin intervention (Figures 5E,F), consistent with the *in vivo* results, with the strongest effect observed in the high emodin dose group.

### 3.6 Emodin affects expression of miR-490-3p and HMGA2 in NRK-52E cells

The expression levels of miR-490-3p in NRK-52E cells treated with TGF-β1 and/or emodin were detected by Real-time PCR, and the protein and mRNA expression levels of HMGA2 were detected by western blot analysis and Real-time PCR. TGF-β1 treatment significantly down-regulated the expression of miR-490-3p mRNA in the NRK-52E cells (Figure 5C), but up-regulated HMGA2 protein and mRNA expression (Figures 5D–F). Compared with the TGF-β1 group, the expression level of miR-490-3p was significantly increased, while the expression level of HMGA2 was decreased in the NRK-52E cells after emodin intervention, with higher doses showing greater effects (Figures 5C–F). These results suggest that emodin can reverse the TGF-β1-induced decrease in miR-490-3p expression in NRK-52E cells.

### 3.7 MiR-490-3p/HMGA2 axis are involved in TGF-β1-induced fibrotic effect in NRK-52E cells

**FIGURE 6:**
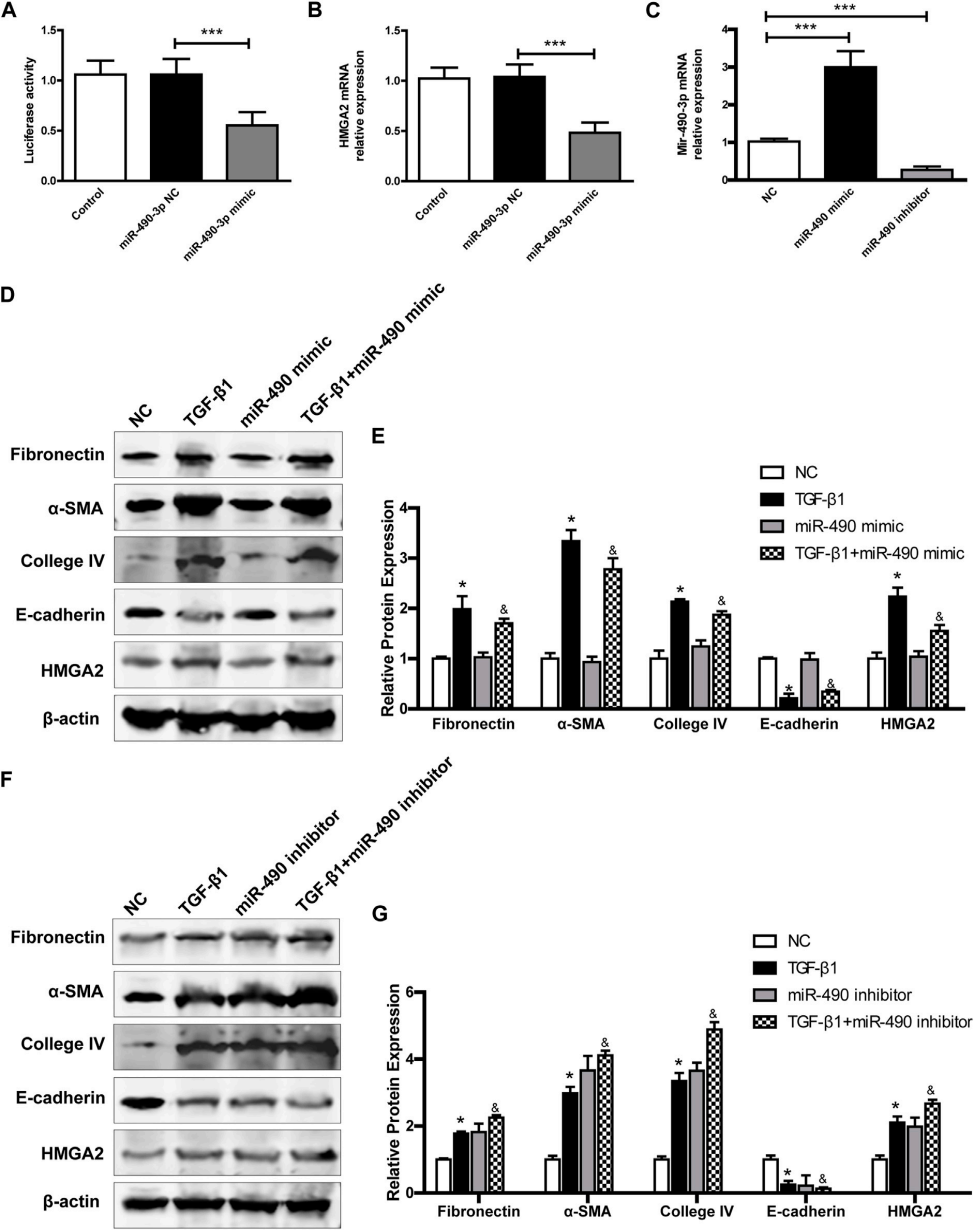
MiR-490-3p/HMGA2 axis are involved in TGF-β1-induced fibrotic effect in NRK-52E cells. HEK293 cells were co-transfected with a NC or miR-490-3p mimic. After 48 h, **(A)** dual luciferase reporter assay and **(B)** Real-time PCR were performed in control, miR-490-3p NC, and miR-490-3p mimic groups to evaluate the luciferase activities and gene level of HMGA2. NRK-52E cells were transfected with miR-490 mimic, NC, or inhibitor for 48 h, and then treated with or without TGF-β1 for 24 h. The gene levels of **(C)** miR-490-3p were evaluated by Real-Time PCR. **(D)** and **(F)**, the protein expressions of Fibronectin, α-SMA, Collagen IV, E-cadherin, and HMGA2 in miR-490-3p overexpression and inhibition experiment, respectively. **(E)** and **(G)**, statistical analyses of relative protein expression in each group. Data were shown as mean ± SD (n = 6). *compared with control, ^&^compared with TGF-β1. *p < 0.05, ***p < 0.001. ^*&*^ *p <* 0.05. TGF-β1, transforming growth factor-β1; HMGA2, high mobility protein A2; α-SMA, alpha-smooth muscle protein.

To verify whether HMGA2 is a potential target of miR-490-3p. We performed dual-luciferase reporter experiments. The HEK293 cells were transfected with miR-490-3p mimics and NCs. As shown in Figure 6A, the miR-490-3p mimic markedly inhibited luciferase activity of the HMGA2-5’UTR reporter, and Real-time PCR showed that, compared to the NC group, HMGA2 was down-regulated after miR-490-3p overexpression (Figure 6B). These results suggest that HMGA2 was targeted by miR-490-3p.

We further investigated the effects of miR-490-3p overexpression and inhibition on HMGA2 and TGF-β1-induced fibrosis in NRK-52E cells. Transfection efficiency was determined by Real-time PCR. As shown in Figure 6C, miR-490-3p expression was increased in the mimic group and decreased in the inhibitor group compared with the NC, indicating that the cells were successfully transfected with miR-490-3p mimic or inhibitor. Western blot analysis showed that TGF-β1 stimulation significantly up-regulated the expression levels of Fibronectin, α-SMA, and Collagen IV, but decreased the expression level of E-cadherin in the NRK-52E cells (Figures 6D–G). Compared with the TGF-β1 group, the miR-490-3p mimic group showed a decrease in the expression levels of Fibronectin, α-SMA, Collagen IV, and HMGA2 proteins but an increase in the expression level of E-cadherin protein (Figures 6D,E), while the miR-490-3p inhibitor group showed an increase in the expression levels of Fibronectin, α-SMA, Collagen IV, and HMGA2 proteins but a decrease in the expression of E-cadherin protein (Figures 6F,G). These data suggest that miR-490-3p is a potent regulator of HMGA2 involved in the regulation of fibrotic effects on NRK-52E cells.

### 3.8 Emodin reduces renal fibrosis by regulating miR-490-3p/HMGA2 axis

**FIGURE 7:**
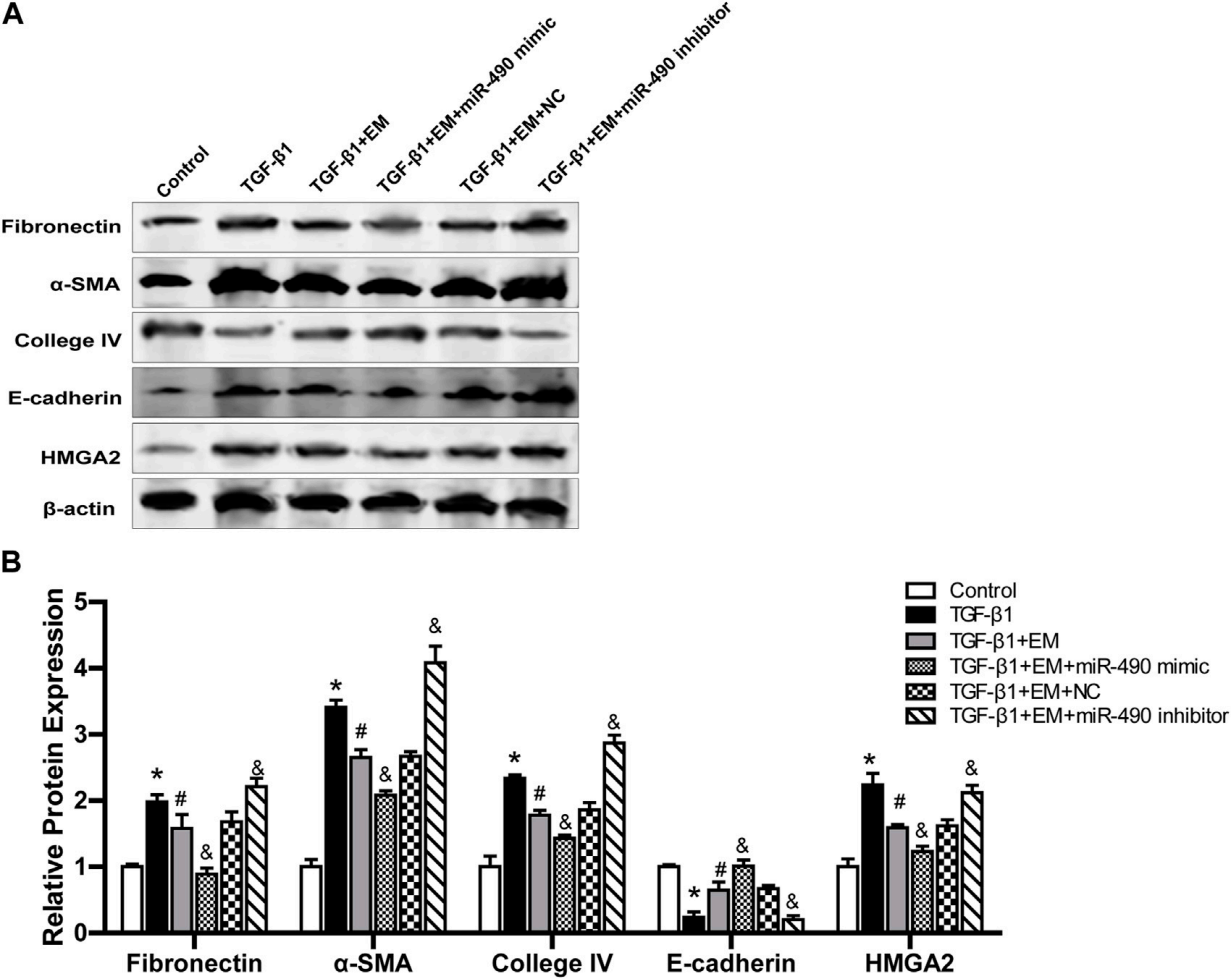
Emodin reduces renal fibrosis by regulating miR-490-3p/HMGA2 axis. MiR-490-3p mimic, miR-490-3p inhibitor, and NC groups of NRK-52E cells were transfected for 48 h, and then treated with or without emodin for 24 h, and finally exposed to TGF-β1 for 24 h. **(A)** Representative blots of Fibronectin, α-SMA, Collagen IV,TGF-β1, and E-cadherin in each group, and **(B)** statistical analyses of relative protein expression. Data were shown as mean ± SD (n = 6). *compared with control, ^#^compared with TGF-β1, ^&^compared with TGF-β1+EM + NC. *p < 0.05, ^*&*^ *p <* 0.05, ^#^ *p <* 0.05. TGF-β1, transforming growth factor-β1; EM, emodin; HMGA2, high mobility protein A2; α-SMA, alpha-smooth muscle protein.

We further studied the regulatory role of miR-490-3p and HMGA2 in the anti-renal fibrotic effects of emodin. Results showed that TGF-β1 stimulation up-regulated Fibronectin, α-SMA, Collagen IV, and HMGA2 protein expression and decreased E-cadherin protein expression in the NRK-52E cells. Emodin treatment significantly alleviated the increase in Fibronectin, α-SMA, Collagen IV, and HMGA2 proteins and decline in E-cadherin protein. Compared with the TGF-β1 +EM + NC group, the TGF-β1 + EM + miR-490-3p mimic group showed a decrease in the Fibronectin, α-SMA, Collagen IV and HMGA2 protein levels and an increase in the E-cadherin protein level, while the TGF-β1 + EM + miR-490-3p inhibitor group showed an increase in the Fibronectin, α-SMA, Collagen IV, and HMGA2 protein levels and a decrease in the E-cadherin protein level (Figure 7). These results suggest that miR-490-3p mimics enhance the protective effects of emodin on TGF-β1-induced NRK-52E cells, while miR-490-3p inhibitors eliminate these protective effects. Thus, emodin may inhibit the HMGA2-dependent signaling pathway and reverse renal fibrosis by up-regulating miR-490-3p expression in NRK-52E cells.

## 4 Discussion

Renal fibrosis is known as the major pathological mechanism of CKD (Nogueira et al., 2017). Rhubarb, a traditional herbal medicine, is widely used in China to treat CKD (Wang et al., 2012). As the active ingredient in rhubarb, emodin exhibits anti-renal fibrosis activity through a variety of ways. Ma et al. reported that emodin ameliorates renal fibrosis by down-regulating TGF-β1 and Smurf two expression (Ma et al., 2018). Liu et al. reported that emodin alleviates epithelial-to-mesenchymal transition (EMT) by activating bone morphogenic protein (BMP)-7-mediated autophagy in renal fibrosis (Liu et al., 2021). Consistently, our research confirmed that emodin is an anti-renal fibrosis agent. Briefly, using a UUO mouse model, we found that emodin increased body weight, decreased blood creatinine, urea nitrogen, and uric acid levels, reduced renal pathological damage, and attenuated renal tissue inflammation and fibrosis in UUO mice. *In vitro*, using TGF-β1-induced NRK-52E cells, we found that miR-490-3p targeting HMGA2 was involved in renal fibrosis, and further showed that emodin *via* regulation of miR-490-3p/HMGA2 and ameliorates renal fibrosis.

The role of inflammation in the pathogenesis and progression of CKD has been recognized since the late 1990s. Recent studies have also confirmed that inflammation and the inflammatory response can alter or interfere with intrarenal microcirculatory regulation and perfusion distribution and can induce renal damage and promote disease progression. Persistent, low-grade inflammation is now considered a distinguishing feature of CKD and is associated with all-cause mortality in patients (Lv et al., 2018a; Mihai et al., 2018). Hirudin is reported to suppress fibrosis in renal tissues and renal tubular epithelial cells by inhibiting inflammation (Xie et al., 2020). Therefore, treating or preventing underlying inflammation may improve CKD prognosis. The anti-inflammatory effects of emodin have been confirmed in various disease studies. Notably, emodin has been shown to significantly down-regulate inflammatory factors, such as TNF-α, MCP-1, IL-1β, and IL-6, *via* regulation of the nuclear factor kappa-light-chain-enhancer of activated B cells (NF-κB) and p38 mitogen-activated protein kinase (MAPK) pathways (Zhu et al., 2013; Xia et al., 2019; Zang et al., 2020). In our study, emodin was selective for the regulation of inflammatory factors in the UUO model, down-regulating TNF-α, MCP-1, and IL-1β, but with no significant effect on IL-6. The result is different from previous results reported for IL-6 regulation by emodin used in other models, it may be due to the different pathophysiological processes of the models.

Fibrosis is a result of tissue repair and becomes dysregulated after many types of tissue damage (Rockey et al., 2015). The fact that changes in fibrosis are linked to various diseases in various organ systems, including heart, kidneys, liver, skin or any other body organ. A variety of cellular and molecular signaling mechanisms are involved in the pathogenesis of fibrosis (Alqudah et al., 2020). Overproduction of cytokines, chemokines, growth factors, extracellular matrix proteins, and loss of normal organ structure and function are common features of fibrosis in organs (Weiskirchen et al., 2019). The ability of fibrosis to resolve may depend on the organ involved, the nature of the injury stimulus, and host-specific factors. The liver stands out from all other tissues with its strong regenerative capacity. In metabolic liver disease, lifestyle changes and bariatric surgery can cause histological fibrosis to regress (Vilar-Gomez et al., 2015). The cardiac parenchymal cells are muscle cells (cardiomyocytes) displaying a very limited regenerative capacity (Zeisberg and Kalluri, 2013). Some researchs on diabetic cardiomyopathy that controlling autophagy and lowering cardiomyocyte apoptosis *via* a variety of routes can prevent cardiomyocyte fibrosis (Zhang et al., 2021; Xue et al., 2022). The kidney has a complex structure, and renal fibrosis is manifested by the presence of glomerulosclerosis, vascular sclerosis and tubulointerstitial fibrosis. Because existing knowledge about the progression of renal fibrosis is extremely complex, preventing or even eliminating renal fibrosis is difficult (Nogueira et al., 2017). TGF-β is considered as one of the common master switches for the induction of the fibrotic program during chronic phases of inflammatory diseases in many organs and tissue. Many evidence suggests that miRNAs are both downstream effectors of TGF-β-dependent renal fibrosis and upstream regulators of TGF-β-dependent signaling pathways (Lv et al., 2018b). Therefore, the regulation of miRNA expression may be as a promising therapeutic strategy for the treatment of CKD.

Various studies have confirmed that HMGA2 is a key factor in the development of EMT. Notably, TGF-β1 is a potent fibrogenic factor that can mediate EMT through the regulatory effects of HMGA2 (Hills and Squires, 2010; Iwano, 2010; Wang et al., 2016)and mediate the development of renal fibrosis through different signaling pathways (Hills and Squires, 2010; Iwano, 2010). EMT is an important mechanism of renal fibrosis (Lovisa et al., 2016). Although the role of EMT in renal fibrosis has been questioned (Kriz et al., 2011), recent evidence suggests that EMT in renal tubular epithelial cells promotes renal fibrosis (Grande et al., 2015; Lovisa et al., 2015) and inhibition of EMT improves renal fibrosis (Sun et al., 2018; Wang et al., 2020; Nagavally et al., 2021). Accumulating evidence also suggests that specific miRNAs play important roles in CKD progression, and that miR-490-3p targeting HMGA2 is implicated in the metastasis and proliferation of multiple tumors (Liu W. et al., 2015; Zhang et al., 2019). Previous sequencing results showed that miR-490 was the most significant down-regulated miRNAs after UUO surgery (Chung et al., 2010; Qin et al., 2011). Our pre-experiment verified the regulatory effects of emodin on the top five miRNAs, including miR-490-3p, miR-137-3p, miR-208-3p, miR-429-3p, and miR-200a-3p, which reported to be significantly decreased in UUO mice (data not shown), and found that emodin had the best regulatory effects on elevating the expression of miR-490-3p. In this study, we found that miR-490-3p was negatively correlated with HMGA2 expression in UUO mice. Using dual luciferase reporter gene assay, we also verified that miR-490-3p exhibited a targeting relationship with HMGA2. Further experiments found that miR-490-3p mimic-inhibited fibrosis in NRK-52E cells was reversed by silencing the expression of HMGA2, and miR-490 inhibitor promoted fibrosis in NRK-52E cells by overexpressing HMGA2. These results suggest that miR-490-3p inhibits fibrosis in NRK-52E cells by down-regulating HMGA2-mediated expression. MiR-490 is also known to have tumor suppressive effects in many cancer types, preventing cancer cells from acquiring a mesenchymal phenotype by modulating EMT, thus inhibiting proliferation and metastasis (Vinchure and Kulshreshtha, 2021). MiR-490-3p is also involved in silicon-induced pulmonary fibrosis by targeting TGFBR1 modulators (Cheng et al., 2021). These findings support our results suggesting that miR-490-3p exerts a protective effect on renal fibrosis. Finally, to identify the underlying molecular mechanism, we detected the effects of emodin on the miR-490-3p/HMGA2 signaling pathway. Results indicated that the miR-490-3p mimic enhanced the protective effects, while the miR-490-3p inhibitor abrogated the protective effects of emodin on TGF-β1-induced NRK-52E cell fibrosis. These findings suggest that emodin alleviates renal fibrosis by up-regulating miR-490-3p. However, limitations of the present study should be considered, the downstream signaling pathways need to be further evaluated in future studies.

In conclusion, emodin increased miR-490-3p expression, inhibited HMGA2 expression, and blocked TGF-β1-induced fibrosis, thereby exerting significant nephroprotective effects and ameliorating UUO-induced renal injury in CKD mice. Therefore, miR-490-3p may be a new drug target for emodin to prevent HMGA2-dependent signaling pathway fibrosis, with potential implications in CKD pathology.

## Data Availability

The raw data supporting the conclusions of this article will be made available by the authors, without undue reservation.
